# Identifying and Screening At-Risk Patients for Hepatitis Delta Virus: A Case Report

**DOI:** 10.7759/cureus.33660

**Published:** 2023-01-11

**Authors:** Bhavana Tetali, Brianna Kuperus, Nikhilesh Mazumder

**Affiliations:** 1 Department of Internal Medicine, University of Michigan, Ann Arbor, USA; 2 Department of Internal Medicine, Division of Gastroenterology and Hepatology, University of Michigan, Ann Arbor, USA

**Keywords:** hepatitis delta virus, screening, viral, hepatitis, hbv, hdv

## Abstract

Hepatitis Delta Virus (HDV) is associated with one of the most severe forms of viral hepatitis, with rapid progression to cirrhosis and hepatocellular carcinoma. While HDV was thought to be uncommon in the United States, recent data shows that its prevalence may be significantly higher than formerly acknowledged. Early identification of HDV is critical since approved therapeutic options are only available for compensated patients. All patients with a reactive hepatitis B virus surface antigen should undergo HDV screening, especially those with additional risk factors, including migration from an endemic area, immunosuppressed patients, hemodialysis patients, gay and bisexual men, persons who inject drugs, and persons employed in healthcare or public safety professions.

## Introduction

Hepatitis Delta Virus (HDV) is associated with one of the most severe forms of viral hepatitis, with rapid progression to cirrhosis and hepatocellular carcinoma [1]. HDV is a defective RNA virus that can only infect individuals with hepatitis B virus (HBV) infection. Despite having been discovered over four decades ago, the prevalence of HDV remains unclear, with recent estimates ranging from 12 million to 72 million cases worldwide [2]. While HDV was thought to be uncommon in the United States, recent data shows that its prevalence may be significantly higher than formerly acknowledged [3]. Given these findings and the severe sequelae of HDV infection, careful consideration should be given to the diagnosis and management of HDV. We present a patient with chronic HBV infection who underwent a laparoscopic cholecystectomy, developed postoperative complications and was ultimately diagnosed with HDV superinfection. This case identifies risk factors for HDV, reviews the pathophysiology of the virus, and explores current therapeutic options.

## Case presentation

A recently emigrated 29-year-old Afghani male with treatment-naive chronic HBV presented to an outside facility with generalized abdominal pain, nausea, and vomiting. He was otherwise healthy and denied alcohol or recreational drug use. He underwent laparoscopic cholecystectomy for symptomatic cholelithiasis and developed significant postoperative abdominal distension. A computerized tomography (CT) scan was performed at the outside facility, which showed a large volume of free fluid in the abdomen. Due to concern over a large bile leak, a diagnostic laparoscopy was performed that was notable for intact surgical clips at the cystic duct. Four liters of fluid were drained, and two Jackson-Pratt surgical drains were placed in the gallbladder fossa, which continued to drain up to two liters of fluid daily. Due to the large volume of fluid output from the surgical drains over the next week and an equivocal diagnostic work-up for bile leak, including an endoscopic retrograde cholangiopancreatography (ERCP), the patient was transferred to our hospital for further evaluation.

Upon transfer, the following laboratory testing was performed (Table 1).

**Table 1:** Laboratory data AST: aspartate aminotransferase; ALT: alanine transaminase; HBV DNA PCR: hepatitis B virus DNA polymerase chain reaction; HBV: hepatitis B virus; IgG: immunoglobulin G; IgM: immunoglobulin M

Laboratory test	Value	Reference range
AST	583 U/L	< 34 U/L
ALT	452 U/L	10 - 49 U/L
Alkaline phosphatase	88 U/L	40 - 116 U/L
Total bilirubin	1.8 mg/dL	0.2 - 1.2 mg/dL
Albumin	2.8 g/dL	3.5 - 4.9 g/dL
HBV DNA PCR	< 10 IU/mL	10 - 1,000,000,000 IU/mL
HBV surface antibody	Non-reactive	N/A
HBV surface antigen	Reactive	N/A
HBV core antibody IgG	Reactive	N/A
HBV core antibody IgM	Reactive	N/A
HBV e antibody	Reactive	N/A
HBV e antigen	Non-reactive	N/A
Hepatitis A antibody IgM	Non-reactive	N/A
Hepatitis C antibody	Non-reactive	N/A
Hepatitis E antibody IgM	Non-reactive	N/A
HIV antigen antibody	Non-reactive	N/A
Cytomegalovirus DNA	< 50 IU/mL	50 - 156,000,000 IU/mL
Epstein-Barr virus DNA	< 501 IU/mL	501-10,000,000 IU/mL
Anti-smooth muscle antibody	Negative	N/A
Antinuclear antibody	Negative	N/A
Alpha-1 antitrypsin S mutation	Not detected	N/A
Alpha-1 antitrypsin Z mutation	Not detected	N/A
Ceruloplasmin	6 mg/dL	16 - 36 mg/dL
Copper 24-hour, urine	20.5 ug	0.0 - 55.0 ug
Serum-ascites albumin gradient (SAAG)	1.4 g/dL	N/A
Ascites protein	< 2.0 gm/dL	N/A
Ascites bilirubin	0.2 mg/dL	N/A

**Figure 1:**
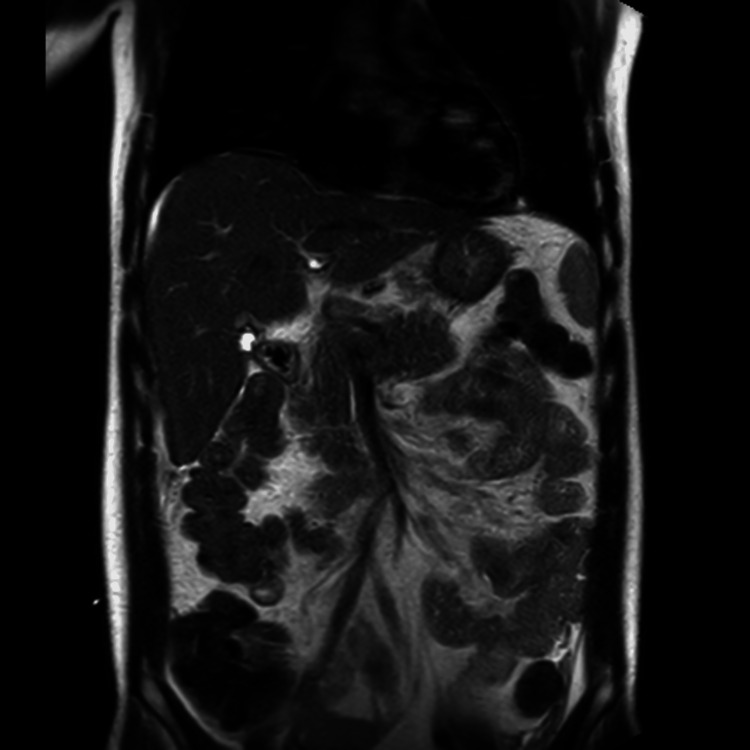
MRCP showing no evidence of postoperative biliary complications but revealing a mildly nodular liver surface

A magnetic resonance cholangiopancreatography (MRCP) was performed. It did not demonstrate a biliary cause for abdominal fluid and showed a mildly nodular liver surface (Figure 1).

**Figure 2:**
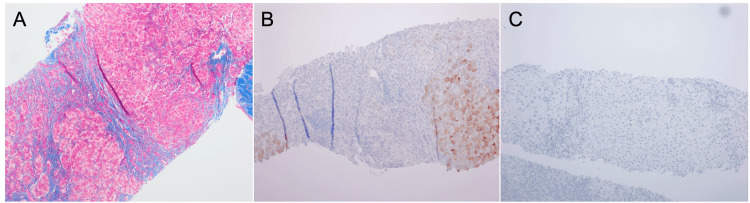
Cirrhosis with hepatitis B virus in the inactive carrier state as revealed by a liver biopsy (A) The background shows markedly active chronic hepatitis consistent with cirrhosis, confirmed with the trichrome stain; (B) immunohistochemical stains demonstrate patchy staining with HBV surface antigen; (C) negative for HBV core antigen

A liver biopsy was subsequently performed, with pathology showing cirrhosis with markedly active chronic hepatitis and immunohistochemical stains demonstrating patchy staining with hepatitis B surface antigen and being negative for hepatitis B core antigen (Figure 2). The patient was found to be positive for HDV antibodies, confirming a diagnosis of cirrhosis caused by HDV superinfection.

The patient had his drains removed and was treated with oral diuretics. He was discharged on diuretics and entecavir for the treatment of HBV infection. Diuretic uptitration is ongoing, with plans to enrol in a trial in the future depending on clinical status.

## Discussion

HDV is transmitted through broken skin or through contact with infected blood and blood products, similar to the transmission routes of HBV. HDV can be acquired simultaneously with HBV as a coinfection, resulting in acute hepatitis that resolves spontaneously in nearly 80% of patients [4]. Alternatively, HDV can be acquired as a superinfection by an individual chronically infected with HBV, resulting in a chronic HDV infection, with nearly 70%-80% of individuals developing cirrhosis or hepatocellular carcinoma within five to 10 years [5,6]. This is the likely cause of cirrhosis in our patient, as cirrhosis in a 29-year-old would not be expected with HBV infection alone.

There is notable heterogeneity in the estimated global prevalence of HDV. While better control of HBV in some parts of the world has reduced the number of individuals susceptible to HDV, improvements in diagnostics over time continue to identify new cases that were previously unknown. Estimates are further complicated by the migration of individuals from endemic countries to areas of the world where HDV is less common. There is a paucity of epidemiologic data on HDV in the United States since there is no national requirement to report HDV cases, making it doubly important for physicians to carefully identify and screen at-risk individuals.

Screening for HDV infection should be considered in all individuals with a reactive HBV surface antigen and especially in patients with elevated ALT levels and low HBV DNA levels [7]. Individuals at higher risk include those who have migrated from Eastern and Southern Europe, the Middle East, Western and Central Africa, and East Asia, where HDV is endemic. Other risk factors include immunosuppressed patients, hemodialysis patients, gay and bisexual men, persons who inject drugs, and persons employed in healthcare or public safety professions [8,9]. Testing should be performed by obtaining total HDV antibodies with confirmation of the diagnosis by serum reverse transcriptase-polymerase chain reaction.

Once diagnosed with HDV infection, treatment should be initiated in patients with active disease as soon as possible, as patients with a shorter duration of infection are thought to have a greater likelihood of suppression [10]. It is vital to identify patients early in their disease since pharmacotherapy is only available to compensated patients. For patients with HDV-related decompensated cirrhosis, a liver transplant is the only available option.

In the United States, pegylated interferon alpha is the only approved pharmacotherapy for compensated HDV infection; however, its mechanism of action and efficacy are still under investigation [11]. Buleviritide, an antiviral that prevents HDV uptake by hepatocytes, is approved by the European Medicines Agency, and lonafarnib, a farnesyl protein transferase inhibitor that prevents HDV assembly within hepatocytes, is an oral agent currently in development [12,13]. Ritonavir, a protease inhibitor, has been used to potentiate the effect of lonafarnib in recent phase 2 studies and is undergoing further evaluation in clinical trials [14]. The endpoint of treatment is suppression of HDV replication after completion of treatment, which generally results in the normalization of ALT levels and decreased inflammation on liver biopsy [15].

## Conclusions

This case highlights the critical importance of prompt identification and screening of patients who are at high risk for HDV. Early recognition of at-risk individuals can aid in making a faster diagnosis, avoiding an extensive diagnostic workup, and providing earlier treatment, which is particularly important as therapeutic options are currently only available for compensated patients. All patients with a reactive HBV surface antigen should undergo HDV screening, especially individuals with additional risk factors, including migration from an endemic area, immunosuppressed patients, hemodialysis patients, gay and bisexual men, persons who inject drugs, and persons employed in healthcare or public safety professions. Once diagnosed with HDV infection, treatment should be initiated as soon as possible to allow for the best likelihood of suppression.

## References

[1] Abbas Z, Afzal R (2013). Life cycle and pathogenesis of hepatitis D virus: a review. World J Hepatol.

[2] Rizzetto M, Hamid S, Negro F (2021). The changing context of hepatitis D. J Hepatol.

[3] Patel EU, Thio CL, Boon D, Thomas DL, Tobian AA (2019). Prevalence of hepatitis B and hepatitis D virus infections in the United States, 2011-2016. Clin Infect Dis.

[4] Miao Z, Zhang S, Ou X (2020). Estimating the global prevalence, disease progression, and clinical outcome of hepatitis delta virus infection. J Infect Dis.

[5] Usai C, Gill US, Riddell AC, Asselah T, Kennedy PT (2022). Review article: emerging insights into the immunopathology, clinical and therapeutic aspects of hepatitis delta virus. Aliment Pharmacol Ther.

[6] Rizzetto M, Verme G, Recchia S (1983). Chronic hepatitis in carriers of hepatitis B surface antigen, with intrahepatic expression of the delta antigen. An active and progressive disease unresponsive to immunosuppressive treatment. Ann Intern Med.

[7] Ahn J, Gish RG (2014). Hepatitis D virus: a call to screening. Gastroenterol Hepatol (N Y).

[8] Chen LY, Pang XY, Goyal H, Yang RX, Xu HG (2021). Hepatitis D: challenges in the estimation of true prevalence and laboratory diagnosis. Gut Pathog.

[9] Da BL, Rahman F, Lai WC, Kleiner DE, Heller T, Koh C (2021). Risk factors for Delta hepatitis in a North American cohort: who should be screened?. Am J Gastroenterol.

[10] Samiullah S, Bikharam D, Nasreen Nasreen (2012). Treatment of chronic hepatitis delta virus with peg-interferon and factors that predict sustained viral response. World J Gastroenterol.

[11] Yardeni D, Heller T, Koh C (2022). Chronic hepatitis D- what is changing?. J Viral Hepat.

[12] Jachs M, Schwarz C, Panzer M (2022). Response-guided long-term treatment of chronic hepatitis D patients with bulevirtide-results of a "real world" study. Aliment Pharmacol Ther.

[13] Dietz CA, Cornberg M (2022). Lonafarnib-a new member of the Delta Force?. Hepatology.

[14] Yurdaydin C, Keskin O, Yurdcu E (2022). A phase 2 dose-finding study of lonafarnib and ritonavir with or without interferon alpha for chronic delta hepatitis. Hepatology.

[15] Terrault NA, Lok AS, McMahon BJ (2018). Update on prevention, diagnosis, and treatment of chronic hepatitis B: AASLD 2018 hepatitis B guidance. Hepatology.

